# Perceptions of the Learning Environment by Undergraduate Medical Students at Government Medical College, Datia: A DREEM Study

**DOI:** 10.7759/cureus.103074

**Published:** 2026-02-06

**Authors:** Vartika Mishra, Santosh Jayant, Kostubh Parihar, Arjun Singh

**Affiliations:** 1 Department of Pathology and Laboratory Medicine, Ruxmaniben Deepchand Gardi Medical College, Ujjain, IND; 2 Department of Pathology and Laboratory Medicine, Amaltas Institute of Medical Science, Dewas, IND; 3 Medicine, Government Medical College, Datia, IND; 4 Department of Pathology, Government Medical College, Datia, IND

**Keywords:** dreem score, learning, medical, perception, teachers

## Abstract

Introduction: The quality of the educational environment significantly impacts how a medical student is going to work in the future as a doctor. The Dundee Ready Educational Environment Measure (DREEM) is a validated tool for assessing this environment. In Madhya Pradesh, India, newer medical colleges have been established, but their educational climates are under-evaluated.

Objective: To evaluate the perceptions of the educational environment among undergraduate medical students at Government Medical College, Datia, using the DREEM questionnaire.

Methods: A descriptive, cross-sectional study was conducted using the 50-item DREEM questionnaire distributed electronically to undergraduate medical students across six batches (2018-2023). Data from 193 respondents (98 male, 95 female) were analysed using descriptive statistics, independent samples t-tests (for gender comparison), and one-way ANOVA (for batch comparison). Internal consistency was assessed using Cronbach's alpha.

Results: The overall mean DREEM score was 128.7 ± 6.40 out of 200, indicating a more positive than negative perception of the environment. The tool demonstrated excellent internal consistency (Cronbach's α = 0.93). Subscale analysis revealed relatively lower scores for Students’ Perception of Teachers (SPT; 27.9 ± 3.01 / 44) and Students’ Social Self-Perception (SSSP; 16.1 ± 2.73 / 28). No significant difference was found between genders in total DREEM scores (p=0.64). However, a statistically significant difference was observed across academic batches (F(5, 187) = 4.05, p=0.002), with the 2019 cohort reporting significantly lower scores than the 2021 and 2022 cohorts.

Conclusion: Students at GMC, Datia, generally perceive their learning environment positively. However, the study identifies critical needs for improvement in social support systems and a shift from teacher-centered, factual learning toward student-centered methodologies.

## Introduction

The educational environment within a medical institution is a foundational pillar determining student learning outcomes, professional identity formation, and the ultimate quality of healthcare delivery [1]. A supportive educational environment is strongly correlated with enhanced academic performance and student well-being, while a perceived negative environment often predicts higher rates of psychological distress and attrition [2]. To quantify this complex construct, the Dundee Ready Educational Environment Measure (DREEM) instrument has emerged as a global diagnostic tool [3]. The rapid expansion of medical education in India, characterized by the establishment of new government medical colleges in underserved districts like Datia, presents a unique challenge in maintaining educational quality amidst potential resource constraints [4]. This study aims to address the critical knowledge gap regarding how students in newly established regional colleges in Central India perceive their training environment [5].

## Materials and methods

This descriptive, cross-sectional study was conducted at Government Medical College, Datia, targeting all undergraduate medical students (MBBS) enrolled from the 2018 to 2023 academic cohorts. Using a census approach, the 50-item DREEM questionnaire was distributed electronically via a secure institutional link over a three-week period between May and December 2024 [6]. The data collection occurred uniformly across all cohorts rather than in staggered phases. The survey was intentionally timed during a mid-semester period, distant from both university examinations and major clinical rotation shifts. This strategy was employed to ensure that responses reflected the students' enduring perceptions of the institutional atmosphere rather than acute situational stress or "exam-period" fatigue. The DREEM is a non-proprietary, validated instrument that is free for academic use; therefore, no formal license was required for this study [1,6]. The instrument consists of 50 items rated on a 5-point Likert scale ranging from 0 (Strongly Disagree) to 4 (Strongly Agree). Based on the standardized scoring system developed by Roff et al. [6], the maximum possible score is 200. Total scores are interpreted as follows: 0-50 (Very Poor), 51-100 (Many Problems), 101-150 (More Positive than Negative), and 151-200 (Excellent) [7,8]. These 50 items are further categorized into five distinct subscales: Students’ Perception of Learning (12 items), Students’ Perception of Teachers (11 items), Students’ Academic Self-Perception (8 items), Students’ Perception of Atmosphere (12 items), and Students’ Social Self-Perception (7 items) [9]. To ensure accuracy, nine negatively phrased items (items 4, 8, 9, 17, 22, 25, 35, 39, and 48) were reverse-scored before data analysis to maintain directional consistency. Data were analyzed using SPSS Version 25.0. In addition to descriptive statistics, independent samples t-tests, and one-way ANOVA with Tukey's post-hoc tests [10], we calculated effect sizes (η²) for the observed differences across academic batches to provide a more robust interpretation of the magnitude of these differences beyond mere statistical significance (p < 0.05). Ethical approval was secured from the Institutional Ethics Committee of GMC, Datia (Approval 40/patho/IECBMHR/GMC/2023).

## Results

A total of 193 undergraduate medical students participated in the study. The cohort was nearly balanced by gender, with 98 males (50.8%) and 95 females (49.2%). The mean age of the participants was 21.8 ± 2.4 years. Representation across academic years varied, with the 2022 batch comprising the largest proportion of respondents (n=101, 52.3%). The detailed demographic distribution is summarized in Table 1.

**Table 1:** Participant demographics (N=193)

Category	Frequency (n)	Percentage (%)
Male	98	50.8%
Female	95	49.2%
Mean SD	21.8 ± 2.4	-
2018	9	4.7%
2019	18	9.3%
2020	10	5.2%
2021	28	14.5%
2022	101	52.3%
2023	27	14.0%

The item-wise analysis of the DREEM reveals specific areas of strength and concern within the institution (Table 2) [6]. Items with a mean score ≥3.5 are considered true strengths, while items with a mean score ≤ 2.0 are identified as problem areas requiring attention [1,11]. The study identified key institutional strengths, as students felt the teachers were knowledgeable (Item 2: 3.3 ± 0.6), expressed confidence about passing the academic year (Item 10: 3.2 ± 0.7), and believed that faculty were well-prepared for their classes (Item 40: 3.0 ± 0.8) [11]. However, areas of concern were also noted, as significant weaknesses were identified in the pedagogical approach and student welfare systems [12].

**Table 2:** Mean scores and standard deviations for DREEM questionnaire Items [6,9,11]. DREEM: Dundee Ready Educational Environment Measure

SN	Subscale / Questionnaire Item	Mean (SD)
I.	Students’ Perception of Learning (SPL)	Total: 24.2/48
1	I am encouraged to participate in class.	1.8 (0.9)
7	Teaching is often stimulating.	2.0 (0.9)
13	Teaching is student-centered.	2.1 (0.9)
16	The teaching helps to develop my competence.	2.0 (0.9)
20	The teaching is well focused.	2.0 (0.8)
21	The teaching helps to develop my confidence.	2.0 (1.0)
24	The teaching time is put to good use.	1.9 (0.7)
25	The teaching over emphasizes factual learning.*	2.1 (0.8)
38	I am clear about the learning objectives of the course.	2.0 (0.8)
44	The teaching encourages me to be an active learner.	1.9 (0.8)
47	Long-term learning is emphasized over short-term learning.	2.0 (0.9)
48	The teaching is too teacher-centered.*	2.4 (0.9)
II.	Students’ Perception of Teachers (SPT)	Total: 25.2/44
2	The teachers are knowledgeable.	1.5 (0.6)
6	The teachers are patient with students.	2.0 (0.9)
8	The teachers make fun of their students.*	3.3 (1.1)
9	The teachers are strict and controlling.*	2.6 (1.0)
18	The teachers have effective communication skills.	2.0 (0.9)
29	The teachers provide good feedback to students.	2.1 (0.9)
32	The teachers provide constructive criticism.	2.6 (1.0)
37	The teachers give clear examples.	2.0 (0.8)
39	The teachers get angry in teaching sessions.*	3.0 (1.0)
40	The teachers are well prepared for their classes.	1.9 (0.8)
50	I feel able to ask the questions I want.	2.2 (0.9)
III.	Students’ Academic Self-Perception (SASP)	Total: 16.9/32
5	Previous learning strategies continue to work for me now.	2.3 (0.9)
10	I am confident about passing this year.	1.7 (0.7)
22	I feel I am being well prepared for my profession.	2.1 (0.9)
26	Last year’s work was good preparation for this year.	2.1 (0.8)
27	I am able to memorize all I need.	2.4 (0.9)
31	I have learned a lot about empathy in my profession.	2.1 (0.8)
41	My problem-solving skills are being well developed.	2.1 (0.9)
45	Much of what I learn is relevant to healthcare.	2.0 (0.7)
IV.	Students’ Perception of Atmosphere (SPA)	Total: 27.3/48
11	The atmosphere is relaxed during ward teaching.	2.0 (0.9)
12	This school is well time-tabled.	2.0 (1.0)
17	Cheating is a problem in this school.*	2.8 (1.3)
23	The atmosphere is relaxed during teaching.	2.1 (0.8)
30	Opportunities exist to develop interpersonal skills.	2.2 (0.9)
33	I feel comfortable in teaching sessions socially.	2.1 (0.8)
34	The atmosphere is relaxed during tutorials.	2.1 (0.8)
35	I find the experience disappointing.*	3.0 (1.0)
36	I am able to concentrate well.	2.2 (0.9)
42	The enjoyment outweighs the stress of the course.	2.5 (1.0)
43	The atmosphere motivates me as a learner.	2.2 (0.9)
49	The students irritate and annoy the teachers.*	3.1 (1.0)
V.	Students’ Social Self-Perception (SSSP)	Total: 18.4/28
3	There is a good support system for stressed students.	2.5 (1.1)
4	I am too tired to enjoy this course.*	2.9 (1.1)
14	I am rarely bored in this course.	3.0 (1.1)
15	I have good friends in this school.	2.0 (0.9)
19	My social life is good.	2.2 (1.0)
28	I seldom feel lonely.	2.7 (1.0)
46	My accommodation is pleasant.	2.2 (1.0)

The DREEM questionnaire demonstrated excellent internal consistency in this sample, with a Cronbach’s alpha of 0.93. The overall mean DREEM score of 128.7 ± 6.40 indicates an educational environment that is categorized as "more positive than negative." Relative ranking of the subscales, based on percentage of maximum possible score, reveals that SASP showed the highest relative strength of 68.8% ( score 22.0/32), followed by SPL: 31.7/48 (66.0%), SPA: 31.0/48 (64.6%), and SPT: 27.9/44 (63.4%). However, SSSP: 16.1/28 (57.5%) shows the primary area for improvement (Table 3). This ranking indicates that while students feel academically confident, they perceive significant deficiencies in their social support systems and the quality of teaching.

**Table 3:** DREEM subscale scores and interpretations [6,11] DREEM: Dundee Ready Educational Environment Measure

Subscale	Items	Max Score	Mean score SD	Interpretation
Perception of Learning (SPL)	12	48	31.7 ± 2.92	A more positive perception
Perception of Teachers (SPT)	11	44	27.9 ± 3.01	Moving in the right direction
Academic Self-Perception (SASP)	8	32	22.0 ± 2.28	Feeling more on the positive side
Perception of Atmosphere (SPA)	12	48	31.0 ± 3.27	A more positive atmosphere
Social Self-Perception (SSSP)	7	28	16.1 ± 2.73	Not a good place
Total DREEM Score	50	200	128.7 ± 6.40	More positive than negative

An independent samples t-test revealed no statistically significant difference in the total DREEM scores between male (126.4 ± 20.7) and female (125.1 ± 21.5) students (t(191) = -0.47, p = 0.64). This indicates that gender does not significantly influence the perception of the learning climate at this institution. A one-way ANOVA demonstrated statistically significant differences in total DREEM scores across the six academic cohorts (F(5, 187) = 4.05, p = 0.002) (Table 4) [11,12].

One-way ANOVA showed a statistically significant difference across batches (F(5,187) = 4.0; p = 0.002) (Table 4) [11,12]. The calculated effect size was moderate (η²= 0.098), indicating that approximately 10% of the variance in DREEM scores is attributable to the academic year of the cohort. The 2021 cohort reported the highest scores, while the 2019 cohort reported significantly lower scores than both the 2021 and 2022 cohorts (p = 0.004).

**Table 4:** Comparison of total DREEM scores across academic batches. *Significantly lower compared to 2021 and 2022 batches based on Tukey's HSD post-hoc analysis. DREEM: Dundee Ready Educational Environment Measure; HSD: Honestly Significant Difference

Academic Batch	N	Mean DREEM Score	Standard Deviation (SD)	Statistical Significance
2021	28	132.5	20.4	Highest perceived quality
2022	101	128.7	20.7	-
2023	27	123.7	20.1	-
2020	10	120.2	12.7	-
2018	9	114.6	29.2	Highest variability
2019	8	109.7	15.8	Significantly lower (p<0.05)*

## Discussion

The overall DREEM score of 128.7 reflects a generally positive environment, similar to findings in other Indian medical schools [11,13]. The SSSP subscale yielded the lowest relative score (16.1/28) [6], identifying it as a critical institutional vulnerability. Specifically, items related to loneliness and boredom received lower ratings, which are major precursors to psychological distress and burnout, as noted in previous applications of this tool [14]. Furthermore, students indicated that teaching is heavily teacher-centered and relies on rote memorization [15]. This deficit likely stems from a combination of structural factors, such as the nascent nature of the institution, which may still be developing robust recreational facilities and formal counseling services and cultural contributors, including the high-pressure environment inherent in medical training that can lead to social isolation. Targeted recommendations include the formalization of student mentorship programs and the improvement of on-campus social infrastructure to bolster student well-being. Additionally, continuous faculty development programs are recommended to move toward student-centered methodologies, such as problem-based learning (PBL). Transitioning from traditional didactic lectures to interactive sessions will address the perceived teacher-centeredness. Finally, the significantly lower scores of the 2019 cohort likely reflect the disruptive impact of the COVID-19 pandemic on their foundational training years [12].

Limitations

This study has certain limitations. First, it is a single-center study, limiting the generalizability of the findings to other medical colleges. Second, the study achieved a response rate of 26.8% (193 of 720 students). While the sample was nearly balanced by gender (98 males, 95 females), the distribution was skewed toward the 2022 cohort (n=101, 52.3%). This may introduce a non-response bias if students who did not participate held more extreme (either highly positive or negative) views. However, the consistency of scores within most cohorts suggests that the findings provide a reliable baseline for the institution.

## Conclusions

The educational environment at GMC, Datia, is generally positive. Nevertheless, critical deficiencies in social support and a perceived reliance on teacher-centered factual recall must be addressed. Implementing targeted welfare and faculty development initiatives will be essential for fostering a well-supported future physician workforce.
